# Update on the Management of Surgical Site Infections

**DOI:** 10.3390/antibiotics11111608

**Published:** 2022-11-11

**Authors:** Biagio Pinchera, Antonio Riccardo Buonomo, Nicola Schiano Moriello, Riccardo Scotto, Riccardo Villari, Ivan Gentile

**Affiliations:** Department of Clinical Medicine and Surgery—Section of Infectious Diseases, University of Naples “Federico II”, Via Sergio Pansini 5, 80131 Naples, Italy

**Keywords:** surgical site infections (SSIs), antimicrobial resistance, risk factors, prevention, prophylaxis, treatment, antibiotics

## Abstract

Surgical site infections are an increasingly important issue in nosocomial infections. The progressive increase in antibiotic resistance, the ever-increasing number of interventions and the ever-increasing complexity of patients due to their comorbidities amplify this problem. In this perspective, it is necessary to consider all the risk factors and all the current preventive and prophylactic measures which are available. At the same time, given multiresistant microorganisms, it is essential to consider all the possible current therapeutic interventions. Therefore, our review aims to evaluate all the current aspects regarding the management of surgical site infections.

## 1. Introduction

Surgical site infections (SSIs) are responsible for about 20% of all healthcare-associated infections (HAIs) and at least 5% of patients undergoing a surgical procedure develop a surgical site infection [1,2,3]. The incidence of SSIs is 2–5% in patients undergoing inpatient surgery; however, the number of SSIs is likely to be underestimated given that approximately 50% of SSIs become evident after the patient has been discharged [4,5,6,7]. A surgical site infection is defined as a surgical wound with local signs and symptoms of an infection, with systemic signs of fever or leukocytosis in severe cases. The surgical site infection is defined as “superficial incisional” when it involves only the skin or subcutaneous tissue, “deep incisional” when it involves the fascia and/or muscular layers and “organ/space” when it involves any part of the body exposed or manipulated during a procedure, excluding the previously mentioned layers [8].

Most surgical site infections are caused by the contamination of an incision with microorganisms from the patient’s own microbial flora during surgery, while an infection from an external source after surgery is less common. Surgical site infections can significantly affect the patient’s quality of life. They are associated with significant morbidity and a prolonged hospital stay. Additionally, surgical site infections place a significant financial burden on healthcare professionals. Indeed, in the study by De Lissovoy G. et al., SSIs are associated with a significant economic burden in terms of an extended length of stay and the increased costs of treatment [9]. The financial burden of an SSI is considerable and ranks as the costliest of the HAIs [2]. Increased costs from SSIs are driven by an increased length of stay (LOS), emergency department visits and readmissions. On average, SSIs extend a hospital length of stay by 9.7 days and increase the cost of hospitalization by over USD 20,000 per admission [2]. Because up to 60% of SSIs were estimated to be preventable with the use of evidence-based measures, SSIs have become a pay-for-performance metric and a target of quality improvement efforts [2]. Surgical patients initially seen with more complex comorbidities [10,11] and the emergence of antimicrobial-resistant pathogens increase the cost and challenge of treating SSIs [12,13,14].

The aim of this review was to describe the current status of surgical site infections and the possible and current preventive and therapeutic strategies to address this condition.

## 2. Risk Factors

It is essential to recognize and identify the risk factors for SSIs in order to be able to prevent and manage them. There are various risk factors for surgical site infections: intrinsic, extrinsic and distinguishable, of which these can be modifiable or non-modifiable. Among the intrinsic risk factors, the modifiable ones are glycemia, respiratory disorders, smoking, alcoholism, obesity, immunocompromised, albumin and bilirubin. Those which are not modifiable are: age, recent radiotherapy and history of skin and soft tissue infections [2,15]. Examples of extrinsic risk factors include procedural risk factors (such as emergency and more complex surgeries and wound classification), those attributable to the hospital facility (such as inadequate ventilation, increased traffic in the operating room and the inadequate sterilization of equipment), and intraoperative risk factors (such as the duration of the surgery, blood transfusions, the maintenance of asepsis, the surgical cleaning of the hands and the use of poor quality gloves, hypothermia and poor glycemic control) [2,15,16]. Strategies to decrease SSIs are multimodal and occur across a range of settings under the supervision of numerous providers. Ensuring a high compliance with these risk-reduction strategies is crucial for the success of SSI reduction efforts.

## 3. Prevention and Prophylaxis

The prevention of SSIs is increasingly important because the number of surgical procedures continues to rise [17]. The human and financial costs of treating SSIs are increasing [18]. It is estimated that approximately half of SSIs are deemed preventable using evidence-based strategies [18,19]. Measures can be taken in the pre-, intra- and post-operative phases of care to reduce the risk of infection [20,21].

Nasal swab screening for Methicillin-susceptible *Staphylococcus aureus* (MSSA) and Methicillin-resistant *Staphylococcus aureus* (MRSA) is the key first step in identifying MSSA or MRSA patients [22]. A nasal decolonization in subjects with *Staphylococcus aureus* (*S. aureus*) with nasal Mupirocin and chlorhexidine body wash is a fundamental strategy to reduce the risk of a surgical site infection [22,23]. People identified as carriers of *S. aureus* who used nasal Mupirocin in combination with a chlorhexidine-based bubble bath prior to surgery had fewer surgical site infections caused by *S. aureus* compared to those who did not have surgery. Although cardiac and orthopedic surgery may be considered high risk, decisions should be made consequent to discussions between the surgical and infection control teams who should take into account the patient’s risk factors [23]. Intranasal Mupirocin should be taken the evening before the day of the surgery and twice a day for 5 days post-op in patients colonized with *S. aureus*. The current guidelines do not indicate the optimal timing for a nasal decolonization given the lack of evidence [1]. However, Mupirocin with chlorhexidine can be given from 2 days before surgery to 3 days after surgery [23]. In some cases, intranasal Mupirocin is used twice daily, starting up to 5 days before surgery in *S. aureus*-colonized individuals. Intranasal Mupirocin decreased sternal wound infections from *S. aureus* in 1850 patients [24,25]. Segers et al. [26] reported a reduction in surgical site infections in patients undergoing treatment with topical nasal chlorhexidine for the decolonization. Active *S. aureus* screening, decolonization and customized antimicrobial prophylaxis decreased the occurrence of infections after hip, knee and cardiac surgery [25,26]. The nasal swab screening for MSSA and MRSA, an attempted decolonization and the use of prophylaxis with Vancomycin appear to reduce the rates of SSIs [27]. Paul et al. highlighted the critical role of nasal swab screening for MSSA and MRSA. In fact, with the identification of patients with MSSA or MRSA, the implementation of decolonization and the use of antibiotic prophylaxis with Vancomycin is allowed to reduce the risk of SSIs [27]. There is evidence that decolonization protocols have to take place close to the time of the surgery in order to be effective [28]. Hospitals should evaluate their SSIs and MRSA rates to determine if the implementation of a screening program is appropriate. However, the use of Mupirocin and chlorhexidine has to be implemented rationally in order to reduce the risk of pharmacological toxicity and the risk of antimicrobial resistance [23,29]. However, it should be emphasized that the benefits of nasal screening and decolonization are currently only demonstrated for orthopedic (together with spine surgery) and cardiac surgery patients, while in other cases of surgical specialties, there are no data to support these strategies. Although there is a need for further data, it is believed at present that nasal screening and decolonization can be useful in any surgical setting.

**Figure 1:**
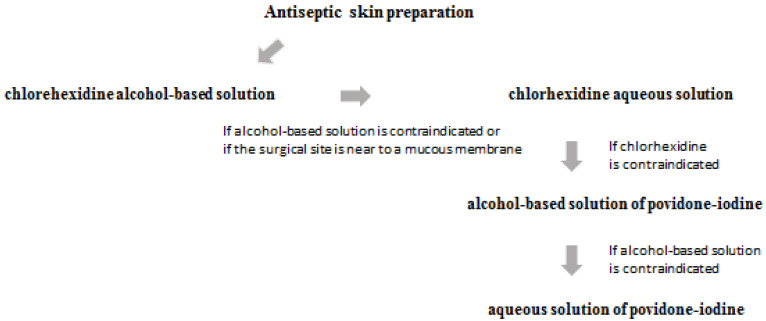
Antiseptic skin preparation.

Another key strategy to reduce the risk of SSIs is the antiseptic skin preparation. The first choice is a chlorhexidine alcohol-based solution, unless contraindicated or if the surgical site is near to a mucous membrane. In the latter case, chlorhexidine aqueous solution is an alternative. If chlorhexidine is contraindicated, an alcohol-based solution of povidone-iodine is the alternative. The use of the aqueous solution of povidone-iodine is the alternative if both an alcohol-based solution and chlorhexidine cannot be used. Compared with the aqueous solution of povidone-iodine, an alcohol-based solution of chlorhexidine was associated with a lower incidence of surgical site infections. The alcohol-based solution of chlorhexidine was found to be cost-effective (Figure 1) [30].

While prevention represents a fundamental strategy, antibiotic prophylaxis is a crucial point in the management of SSIs on the other. Antibiotic prophylaxis is recommended for patients with clean surgery involving the placement of a prosthesis or implant, clean-contaminated surgery and contaminated surgery. The routine use of antibiotic prophylaxis for clean non-prosthetic uncomplicated surgery is not recommended [23]. Prophylaxis consists of giving a single dose of intravenous antibiotic therapy, taking into account the pharmacokinetics of the drug and the timing of the intervention. This dose should be repeated if the duration of the intervention exceeds the half-life of the drug. An antibiotic treatment should be considered if the wound is dirty or infected [23]. To be optimally effective and deliver high tissue levels at the time of the incision, antibiotics should be started within 60 min of the surgical incision [23]. Since Vancomycin and Fluoroquinolones may require 1–2 h of infusion time, they should be started 2 h before the surgical incision. For procedures lasting >2 half-lives of the prophylactic agent (generally surgeries over 4 h), an intraoperative supplementary dose may be required. In the event that the surgery lasts more than 4 h, it is essential to carry out at least a second dose of antibiotics. [23]. A series of pharmacokinetic studies are underway that are evaluating the effective dosages of prophylactic antibiotics in patients with a high body mass index, as this category of patients may probably need higher dosages. [23]. Vancomycin is considered an alternative in patients allergic to or intolerant to β-lactams. The use of vancomycin may be justifiable in centers where the rates of a post-operative infection with MRSA are high or in patients at a high risk of an MRSA infection. It should be emphasized that when choosing Vancomycin due to the possible risk of MRSA, it is possible to consider in addition the use of Cefazolin, in order to ensure an optimal protection against any MSSA. Unlike β-lactams in common use, Vancomycin has no activity against a Gram-negative organism. When Gram-negative bacteria are a concern following specific procedures, it may be necessary or desirable to add a second agent, such as Cefazolin or other Gram-negative agents (e.g., Aminoglycoside, Fluoroquinolone or Aztreonam). In cases where it would not be possible to use neither the b-lactams nor the Vancomycin, 900 mg of Clindamycin iv as a single dose can be used, to be repeated after 6 h if the surgery lasts this long [23]. Prophylaxis does carry risk, e.g., *C. difficile colitis* [23].

Antibiotics should be discontinued at the time of the incision’s closure, except in implant-based breast reconstructions, joint arthroplasty and cardiac procedures where the optimal duration of antibiotic therapy remains unknown. In general, there is no evidence that an antibiotic administration after the incision’s closure decreases the risk of an SSI across a range of procedures, including clean, clean-contaminated and contaminated wound classes. There are several exceptions, namely when the optimal duration of antibiotic prophylaxis is controversial or unknown. The use of single dose prophylaxis is deemed to be adequate for primary augmentation mammoplasties. Contrary to these findings, a large systematic review demonstrated no benefit with antibiotics past 24 h [23]. Similarly, a matched cohort study found no difference in SSIs between patients receiving a single preoperative dose of antibiotics versus an extended postoperative course. Moreover, a recent prospective trial found no benefit by extending prophylaxis beyond 24 h [23]. A systematic review of four randomized controlled trials found no evidence to support postoperative antibiotics (versus a single dose preoperatively) [23].

The use of various topical and local antibiotic therapy options for the reduction in SSIs has been explored across many surgical subspecialties. Overall, there is a lack of high-quality data to support local and topical antibiotic therapy use to decrease the risk of an SSI. These therapies include antibiotic irrigations, topical antimicrobial agents, antimicrobial-impregnated dressings and wound sealants [31]. A recent systematic review found it to be of a possible benefit for their use in joint arthroplasty, cataract surgery, and possibly in breast augmentation and obese patients undergoing abdominal surgery [31]. A meta-analysis concluded that the use of Vancomycin powder at the surgical site was associated with a lower SSI risk for spine surgery. There is inadequate evidence to support the routine use of topical or local antimicrobial agents, although there may be a benefit from specific procedures and patient populations [31].

## 4. Treatment

In view of the therapeutic approach, it is essential to know that the agents responsible and mainly involved in surgical site infections are *S. aureus*, coagulase negative staphylococci, *Enterococcus* species and *E. coli*. At the same time, it is essential to know the local epidemiology and the resistance profiles of the corresponding suspected or identified microorganisms [32]. One of the increasingly growing problems is antimicrobial resistance, in fact, the choice of antibiotic therapy must increasingly take into account multidrug resistance germs.

### 4.1. Available Treatments for Gram-Positive Bacteria: Old and Novel Drugs

The choice of the most appropriate antibiotic for the treatment of Gram-positive infections cannot be based solely on the strain’s susceptibility pattern, but also with regard to the activity against biofilms, the impact on the toxin production, pharmacodynamic parameters associated with an optimal efficacy, the dosage, the mechanism of resistance and the main adverse events [33,34].

*Staphylococcus aureus* represents one of the main responsible agents of the SSIs and it can cause disease due to toxins or superantigens, suppuration, tissue necrosis, vascular thrombosis and bacteremia. It can form biofilms which are responsible for chronic infections and it colonizes some areas of the skin and mucosa (nares, oropharynx and perineal skin) of 40% of the healthy population from where it causes reinfections, contaminates the environment and spreads to other patients. In the cases of staphylococcal infections, the risk of complications and mortality are high [34]. The prevalence of methicillin-resistant Staphylococcus aureus (MRSA) has increased dramatically in recent decades; almost 7% of patients are screen positive for MRSA [35]. Although the incidence of an MRSA infection following a major surgical procedure is estimated to be only 1% overall, an MRSA colonization is associated with worse outcomes and a higher risk of both MRSA–SSI and SSI overall [35,36,37]. The estimated prevalence is <0.1% of the total isolated methicillin-resistant MRSA [38]. In this regard, there are new antibiotic therapies, but clinical trials regarding these antibiotic therapies are scarce. However, some studies have evaluated the potential usefulness of new therapies [39]. Once the species is identified, the early detection of methicillin resistance is necessary, as MRSA is resistant to all β-lactams except for Ceftaroline and Ceftobiprole [40]. Interestingly, the new cephalosporins with activity against MRSA, Ceftobiprole and Ceftaroline, have demonstrated a low inoculum effect. Ceftobiprole has shown a high stability after 24 h of exposure to a high inoculum of a penicillinase-producing S. aureus strain being even more stable than methicillin [41,42]. However, while Ceftaroline is indicated for skin and soft tissue infections, as well as community-acquired pneumonia, Ceftobiprole is currently only indicated for community-acquired and nosocomial pneumonia [43]. For MRSA infections, the highest dose of fifth generation cephalosporins [Ceftaroline 600 mg/8 h and Ceftobiprole 500 mg/8 h] is recommended until reaching the minimum inhibitory concentration (MIC) [43].

As a therapeutic possibility in the on-label mode, Vancomycin remains a potential therapeutic strategy for Gram-positive multi drug resistance (MDR). Vancomycin has a time-dependent bactericidal activity against Gram-positive cocci, slower than that observed with β-lactams. Compared to Vancomycin, Teicoplanin has the advantage of being administered in a single daily dose, with a shorter infusion time, less nephrotoxicity and practically no risk of red man syndrome. However, the intrinsic activity and bactericidal effect are lower than the dose of Vancomycin and the selection of resistant mutants occurs more frequently [44].

Another important therapeutic option for Gram-positive in the setting of SSIs is Daptomycin. It is a lipopeptide active against MSSA, MRSA, methicillin-susceptible *Staphylococcus epidermidis* (MSSE) and methicillin-resistant *Staphylococcus epidermidis* (MRSE) and it has a concentration-dependent bactericidal activity. Daptomycin is active against Gram-positive bacteria, including methicillin-resistant *Staphylococcus aureus* (MRSA) and the on-label indications for its use include complicated skin and skin structure infections (cSSSI). Daptomycin could be very useful in the management of surgical site infections, given its pharmacokinetic characteristics, but further human studies with a histological examination are needed. [45,46]. Most associations of Daptomycin with other antimicrobials, especially with beta-lactams or Fosfomycin, show a synergistic or additive activity [46,47]. The association with Rifampicin significantly increases the activity of Daptomycin against intracellular forms. Daptomycin alone, and especially in association with Linezolid or Rifampicin, is active against *S. aureus* within biofilms. Daptomycin, at 4–6 mg/kg/day, has been compared with Vancomycin, Teicoplanin or Cloxacillin in several randomized studies involving patients with complicated skin and soft tissue SST, bacteremia or endocarditis due to *S. aureus*. The clinical results and microbiological eradication rates in patients receiving Daptomycin have been similar to those obtained with the comparators and the renal toxicity was significantly lower in Daptomycin when compared with Vancomycin [47].

Unlike the aforementioned antibiotics for Gram-positive, Linezolid has bacteriostatic activity and decreases the production of toxins and other virulence factors by *S. aureus* [48]. Although it has an excellent tissue bioavailability, the activity of Linezolid against a microorganism growing in biofilms or intracellularly is limited. Linezolid is a small molecule, with 30% protein binding and amphiphilic properties. These characteristics give Linezolid an excellent diffusion to the surgical site [48]. Most Linezolid associations with other antibiotics are indifferent or, less frequently, antagonistic. In the treatment of SSSTI, Linezolid is superior to Vancomycin in MRSA infections and similar to Cloxacillin in MSSA infections [48].

The new lipoglycopeptides (Dalbavancin and Oritavancin) are potent anti-staphylococcal agents with a long half-life [40]. The mechanism of action, common to glycopeptides class, is the inhibition of the bacterial cell wall synthesis but the presence of an additional lipid side chain anchors these molecules to the cell membrane and thereby concentrates the drug at its site of action, increasing the drugs’ potency relative to their parent glycopeptide. In addition, it has been proposed that the lipid side chain of Oritavancin may act as a detergent-like molecule, causing the partial destabilization of the cell membrane and the loss of the membrane potential. This additional mechanism confers to the Oritavancin activity against a slowly growing microorganism and biofilms. Dalbavancin has long-acting bactericidal activity following a single dose, and it was demonstrated that one large (10 mg/kg) dose of Dalbavancin prevented *S. aureus* regrowth for 120 h, whereas it took four doses of Vancomycin to produce the same effect. The clinical efficacy and safety of Dalbavancin was demonstrated in phase III clinical trials in complicated and uncomplicated skin and skin and soft tissue infections (SSSTI) [49]. The comparator arms included Linezolid, Vancomycin and Cefazolin and Dalbavancin showed a similar efficacy [49]. The accepted doses for SSSTI are a single shot of 1500 mg or 2 doses of 1000 mg and a second one of 500 mg a week apart [49]. For Oritavancin, 2 double-blind, randomized phase III studies demonstrated a similar clinical efficacy and the safety of a 1200 mg single dose of Oritavancin versus Vancomycin in SSSTI [40].

Additionally, the Coagulase-negative staphylococci (CoNS) are responsible for a high number of infections. *Staphylococcus epidermidis* is the most pathogenic species and is isolated in 45–80% of the infections produced by CoNS. They generally cause nosocomial infections. The antibiotic of choice, according to the latest guidelines, is Vancomycin. Although the data are scarce, the Daptomycin, fifth generation cephalosporins, Linezolid or lipoglycopeptides could be acceptable alternatives. It is possible following the same principles as for *S. aureus*.

Among Gram-positive bacteria, *Enterococcus faecalis* and *Enterococcus faecium* are recognized as major causative pathogens of health care-associated infections (HAIs). Most of the enterococcal species are intrinsically resistant to several antimicrobials, such as cephalosporins, aminoglycosides, clindamycin and trimethoprim-sulfamethoxazole [50]. The major problems of these species are their potential multidrug resistant (MDR) nature and ability to form biofilms [51]. *E. faecium* is included in the acronyms of ESKAPE (*Enterococcus faecium, Staphylococcus aureus, Klebsiella pneumoniae, Acinetobacter baumannii, Pseudomonas aeruginosa and Enterobacter* spp.), organisms which are relevant pathogens responsible for HAIs and for which new antibiotics are urgently needed [52]. *E. faecalis* is usually susceptible to penicillin and ampicillin, unlike *E. faecium* [50]. A potential exception can be Ceftaroline and Ceftobiprole, extended spectrum cephalosporins with notable in vitro activity against *E. faecalis*, but not *E. faecium* [40]. There has been an increase in the percentage of Vancomycin-resistant isolates of *E. faecium*, from 10.5% in 2015 to 18.3% in 2019 [53]. The European Committee on Antimicrobial Susceptibility Testing (EUCAST) in its expert rules recommends that Vancomycin-susceptible isolates may be reported to be susceptible to Dalbavancin, Oritavancin or Telavancin [54]. In the case of a Vancomycin resistance, the MIC to these compounds should be determined. A resistance to Daptomycin is uncommon, although it may be developed during treatment [55,56]. EUCAST has not established clinical breakpoints for Daptomycin in enterococci. For EUCAST in this scenario, clinical studies with MIC outcome correlations are needed to define the clinical breakpoints [55]. Monotherapy with β-lactams in sensitive isolates could be sufficient in places of easy access to the antimicrobial [57,58]. As alternatives to the standard regimens, Linezolid or Daptomycin have been used. Daptomycin has also been associated with ampicillin with good results in strains with a decreased susceptibility to ampicillin or even Daptomycin. Other associations tested, either in vitro, in animal trials or reported in clinical cases include two β-lactams (Ampicillin with cephalosporins): Daptomycin with a β-lactam, an aminoglycoside, Fosfomycin or Tigecycline, and Fosfomycin with Ceftriaxone, Gentamicin, Teicoplanin, Rifampicin or tigecycline [57,58].

Despite the increasing antimicrobial resistance regarding Gram-positives in the context of SSIs and nosocomial infections, we have several therapeutic options at our disposal in this setting that allow us to deal with these conditions. Particularly interesting and innovative is the use of long-acting drugs, which could revolutionize the management of these infections. In view of the advent of some drugs such as Fosfomycin, the role of combination therapy in this area is yet to be clarified.

### 4.2. Available Treatments for Gram-Negative Bacteria: Old and Novel Drugs

The emergence of severe infections caused by multi-drug resistant (MDR) Gram-negative microorganisms represents a serious public health concern [59].

The major resistance mechanism among *Enterobacterales* is the production of β-lactamases, in particular, the extended-spectrum β-lactamases (ESBL), the carbapenemases and the AmpC-type β-lactamases. Decreasing the outer membrane permeability and efflux system can also contribute to the resistance. The overuse of carbapenems in turn selected for the emergence and dissemination of carbapenem-resistant *Enterobacterales* (CRE) and of non-fermenting Gram-negative strains resistant to carbapenems [59].

New therapeutic options have arisen in recent years, including ceftolozane/Tazobactam and Ceftazidime/Avibactam. Ceftolozane/Tazobactam has demonstrated an overall higher activity against ESBL producers in vitro studies and was successfully used to treat ESBL infections in clinical studies [60]. A recently published Italian retrospective multicenter study (CEFTABUSE II), including 153 patients with infections due to ESBL-producing *Enterobacterales,* confirmed that ceftolozane/Tazobactam could be a valid option regardless of the approach being empirical or targeted [61]. Clinical success in this study was achieved in 100% of patients treated empirically, while patients who received targeted or rescue therapies were successfully treated in 83.8% and 66.7% of cases, respectively [61]. The ASPECT-NP study also supports the choice of ceftolozane/Tazobactam for the treatment of severe infections by ESBL-producing *Enterobacterales*. In that study, ceftolozane/Tazobactam was found to be comparable to Meropenem in terms of the efficacy for the treatment [62].

Several studies have demonstrated the superiority of Ceftazidime-Avibactam over older therapeutic regimens in treating infections caused by KPC (Klebsiella pneumoniae carbapenemase)-producing CPE (carbapenemase-producing Enterobacteriaceae). Shields et al. demonstrated that Ceftazidime-Avibactam was superior in terms of its efficacy, mortality and clinical cure at 30 days, compared to any other combination regimen used [63]. Italian retrospective data on the compassionate use of Ceftazidime-Avibactam vs. the “best available therapy” in 104 patients showed that 30 days mortality was significantly lower in the Ceftazidime-Avibactam group (36.5% vs. 55.7%, *p* = 0.005) [64]. Meta-analyses, however, showed that monotherapy with Ceftazidime-Avibactam has a similar efficacy compared to combo therapy regimens for the treatment of CRE infections, with a statistically significant difference in terms of the mortality or microbiological eradication [65]. However, Ceftazidime-Avibactam has shown the potential for the selection of resistant mutants, and several reports have documented the potential danger of monotherapy with Ceftazidime-Avibactam in view of this [66].

New drugs active against KPC producers have recently been approved for clinical use, namely Meropenem-Vaborbactam and Imipenem-Relebactam. Vaborbactam is a new non-β-lactam inhibitor of β-lactamases, derived from boronic acid, which inhibits KPC-type carbapenemases and, combined with Meropenem, efficiently protects the antibiotic from hydrolysis by KPC [67]. Imipenem/Relebactam is a new antibiotic based on another non-β-lactam-based inhibitor of the diazabicycloctane family, which is active against KPC-type carbapenemases [68].

Furthermore, *Enterobacterales* can acquire Metallo-β-lactamases (MβLs) genes carried on plasmids by a horizontal gene transfer. The most common acquired MβLs encountered in *Enterobacterales* are VIM (Verona integron-encoded Metallo-β-lactamase), IMP (imipenemase metallo-β-lactamase) and NDM (New Delhi metallo-β-lactamase). Colistin-dependent therapeutic regimens, Aztreonam + Ceftazidime/Avibactam and cefiderocol are the possible therapeutic options [69,70]. Cefiderocol is the first siderophore antibiotic which penetrates the bacterial cell through the iron transporters and is active against most isolates of *Enterobacterales* and Gram-negative non-fermenters, including strains producing β-lactameases of the different classes [71,72].

Among Gram-negative, *Pseudomonas aeruginosa* is considered to be among the most dangerous nosocomial pathogens, especially due to the development of multi-drug resistance (MDR) and pan-drug resistance (PDR) [73]. Multi-resistant strains have increased in recent years and the detection of 15–30% of resistant isolates are not uncommon in some geographical areas [73,74]. The new β-lactam/lactamase inhibitor combinations (BLICs) such as ceftolozane/Tazobactam, Ceftazidime/Avibactam, Imipenem/Relebactam and Cefiderolcol are active against *Pseudomonas aeruginosa*. Ceftazidime/Avibactam is a substrate of *Pseudomonas* efflux pumps. The resistance of ceftolozane/Tazobactam, therefore, has a significantly lower rate than that of Ceftazidime/Avibactam in *P. aeruginosa* isolates with reduced oprD porins and an increased mexB expression (5.1% vs. 25.6%, *p* > 0.025 and 4.3% vs. 34.8%, *p* > 0.022, respectively) [75].

However, there are few data regarding the use of ceftolozane/Tazobactam in the context of surgical site infections. The studies by Rempenault C. et al. and Hassan S. et al. highlighted the efficacy of this drug in orthopedic surgical site infections from *Pseudomonas aerugnosa* MDR [76,77]. In the study of Mora-Guzmán I. et al. fifty patients with a superficial incisional SSI of 50%, a deep incisional SSI of 28% and an organ/space SSI of 70% were treated with Ceftazidime/Avibactam in combination and the global 30-day mortality rate for an intra-abdominal infection was 20% [78,79]. Imipenem/Cilastatina/Relebactam was assessed in the SMART study and in *P. aeruginosa* strains and the addition of Relebactam was shown to restore the activity of Imipenem/cilastatina [68]. The phase 3 RESTORE-IMI 1 trial was a multicentric, randomized, double-blind control aimed at comparing both the efficacy and safety of Imipenem/Relebactam with the combination scheme of Imipenem+Colistin for the treatment of infections in hospitalized patients sustained by Imipenem-resistant pathogens [80,81]. The study showed a similar response for both arms (71% vs. 70% for Imipenem/Relebactam and Imipenem+Colistin, respectively) [81]. Adverse events were lower in the Imipenem/Relebactam arm as opposed to the control arm [16% vs. 31%], including adverse events leading to nephrotoxicity (10% vs. 56%) [80,81]. Meropenem/Vaborbactam acts on AmpC-producing *Pseudomonas aeruginosa*, but no differences were noted between Meropenem/Vaborbactam and Meropenem and *Pseudomonas aeruginosa* with other resistance mechanisms [67]. A recent CREDIBLE-CR study aimed to assess the efficacy and safety of cefiderocol vs. best available therapy (BAT) for the treatment of patients with carbapenem-resistant Gram-negative infections [61]. The study showed a similar clinical success for *Pseudomonas aeruginosa* across the two groups with, the overall clinical success for *P. aeruginosa* being 58% (7/12) for the cefiderocol group vs. 50% for the BAT group (5/10) [61]. Interestingly, clinical success in *Enterobacterales*-induced infections showed a superiority of cefiderocol treatment over BAT, while infections sustained by non-fermenting Gram-negative showed similar clinical success efficacies [61]. There are no data in the literature regarding the use of cefiderocol in the context of surgical site infections, except for sporadic case reports. In particular, we found a case of a patient successfully cured with cefiderocol for a neurosurgical site infection due to extensively resistant *P. aeruginosa*, who had failed a previous treatment based on a combined antimicrobial therapy and right parietal bone excision [79]. Another clinical case involved the treatment of an *Acinetobacter baumannii* extensively drug-resistant (XDR) joint prosthesis infection, treated for 25 days with cefiderocol and tigecycline with a clinical resolution. The clinical case demonstrates that cefiderocol may be useful as a therapy for patients with limited treatment options due to an antimicrobial resistance [82].

Cefiderocol has also demonstrated activity against *Acinetobacter* and is currently the only β-lactam displaying such activity against this microorganism [61]. While Eravacycline has demonstrated some in vitro activity against *A. baumannii* [83], only Colistin and cefiderocol have shown significant activity against this pathogen [61,83]. The cefiderocol treatment reached predefined non-inferiority status in clinical trials, despite a mortality imbalance reported in the CREDIBLE-CR study [61]. The CREDIBLE-CR study for the assessment of the efficacy and safety of cefiderocol vs. the best available therapy (BAT) was designed to enroll patients with infections caused by Gram-negative carbapenem-resistant (CR) pathogens. Mortality at day 28 was 38% in the cefiderocol arm and 18% in the BAT arm. Mortality in the cefiderocol group was not attributed to any specific factors other than a disproportion of patients with shock (26% in the cefiderocol group vs. 6% in the BAT group) and a greater proportion of patients admitted to the ICU (81% in the cefiderocol group vs. 47% in the BAT group) [61].

Among the drugs already in use for many years, in the context of SSIs, tigecycline represents a considerable therapeutic possibility due to its bioavailability and its spectrum of action. Tigecycline is active in vitro against *A. baumannii*. Despite this, most of the publicly available literature concerning the clinical data for tigecycline is retrospective and mainly describes its therapeutic regimens in combination with other molecules. The clinical activity of tigecycline is almost always confirmed, though this is due to its large volume of distribution and low blood concentration [84]. Tigecycline is confirmed as an optimal therapeutic option in cases of surgical site infection, as its spectrum would allow it to cover Gram-positive, including MRSA, Gram-negative and anaerobes. However, in case of a systemic involvement and concomitant infections of the bloodstream, due to its bacteriostatic activity, the choice of this antibiotic should be reconsidered in favor of other therapeutic alternatives, with bactericidal activity [85]. Sulbactam, an old suicidal β-lactamase inhibitor, is active against the bacterium. Its bactericidal activity is attributed to a different mechanism related to its affinity and acylation of Acinetobacter [86]. Sulbactam proved to be the most effective on the mortality outcomes [87]. Furthermore, the meta-analysis performed by Jung et al. suggested that high dose SUL (9 g/day or even higher doses) co-administered with intravenous Colistin was superior to single agent Colistin regimens both in terms of survival and clinical sure [87]. The association combined Fosfomycin/Sulbactam is currently of great therapeutic attraction, despite *Acinetobacter*’s genetic resistance to Fosfomycin as mediated by the effect of its efflux pumps. Lim et al. tested the synergism of FOS/SUL on 50 isolates of carbapenem-resistant *A. baumannii* (CR-AB) strains. A synergistic effect was observed in 74% of cases and no cases of antagonism were reported [88].

Furthermore, the advent of Fosfomycin in recent years has made it possible to evaluate the possibility of combination therapies. There are few studies regarding the use of Fosfomycin iv in cases of surgical site infections, however in the various studies considered, a favorable role of this drug in this setting was highlighted. In particular, in the study by Simonetti O. et al., the synergistic and bactericidal activities of the antimicrobial associations of Fosfomycin with Rifampicin and tigecycline against Enterococcus faecalis, Enterococcus faecium and clinical isolates of methicillin-resistant Staphylococcus aureus (MRSA) are highlighted [89]. The drug combinations showed the highest antimicrobial effects compared to monotherapy [89]. The study showed the efficacy of Fosfomycin combinations [89]. Additionally, in the study by Kusachi et al., the favorable use and efficacy of Fosfomycin in combination for an intra-abdominal abscess refractory treatment was confirmed [90]. Now, although there are few data in the literature regarding the use of Fosfomycin in the context of surgical site infections, due to the pharmacokinetic characteristics of the drug and the data already present, Fosfomycin in combination could represent a valid therapeutic choice for surgical site infections.

Regarding the management of Gram-negative SSIs, although new therapeutic possibilities have arisen in recent years, the problem of antimicrobial resistance looms and does not seem to stop, so much so that the need to seek new therapeutic resources is recognized. In this regard, the importance of the rational management of the current antibiotic therapies and at the same time the importance of antimicrobial stewardship and infection control are underlined.

### 4.3. Future Prospects

In the future, new therapeutic possibilities will be available to face the fight against antimicrobial resistance. In particular, amongst non β-lactams, new therapeutic options for treating ESBL infections include Eravacycline and Plazomicin.

Eravacycline is a synthetic fluorocycline antibacterial agent that is structurally similar to tigecycline, with two modifications at the D-ring of its tetracycline core, and it is approved for the treatment of complicated intraabdominal infections [91]. The in vitro activity is similar to tigecycline but MICs are often two-fold lower than tigecycline [92]. Eravacycline has successfully completed phase three of the clinical trial for the treatment of cIAI (complicated intra-abdominal infections) [93] and it was non inferior to Ertapenem or Meropenem in a phase III, randomized, double-blind clinical trial of patients with appendicitis, cholecystitis, diverticulitis, gastric or duodenal perforation and peritonitis [93]. It could be a suitable candidate for the treatment of cIAI caused by XDR, and even PDR pathogens, such as *Enterobacterales* ESBL, KPC and MBL; *A. baumannii*, taking into account that its spectrum of action also includes *Enterococci*; and MRSA and anaerobes [83]. Therefore, further clinical studies addressing the efficacy of Eravacycline in difficult-to-treat infections is required.

Plazomicin is a semisynthetic aminoglycoside derived from sisomicin. It is active against *Enterobacterales* resistant to β-lactams and other classes of antibacterials and it might have a reduced activity vs. *Enterobacteriaceae* that express efflux pumps or has an impaired cell wall permeability due to closure of the porin. It is not active against *Acinetobacter baumannii*, *Stenotrophomonas maltophilia* and anaerobes, but its activity is variable activity against *Pseudomonas aeruginosa*. It is currently approved for complicated urinary tract infections, including pyelonephritis, caused by aerobic Gram-negative bacilli [83,94].

Additional agents with activity against MβL producers are in advanced stages of the pipeline. These include the novel diazabicyclooctanes that also exhibits an intrinsic antibacterial activity by targeting PBP2 (penicillin-binding proteins2), such as Nacubactam and Zidebactam, developed in combination with Meropenem or Cefepime [95]. Some novel boronate derivates, such as Taniborbactam, which exhibit a broad-spectrum β-lactamase inhibition profile covering also most MβLs, were developed in combination with Cefepime: Cefepime/Taniborbactam, Cefepime/Zidebactam, Cefepime/Enmetazobactam and Meropenem/Nacubactam [96].

Among the future options, Sulbactam/Durlobactam is generating great interest. Durlobactam is a novel serine b-lactamases inhibitor, capable of restoring sulbactam’s activity against resistant *A. baumannii* strains. Seifert et al. highlighted the excellent activity of the combination, comparable to Colistin and better than amikacin, minocycline and Sulbactam alone [97].

The future perspectives of antibiotic therapy represent a concrete future reality and a further support in the fight against antibiotic resistance. However, these resources and hopes have not distracted us from the importance and our goal of reducing resistance rates through the control of infections and antimicrobial stewardship.

## 5. Conclusions

Our review highlights how surgical site infections are increasingly an emerging problem, also taking into account the impact of antimicrobial resistance. We currently have several and new therapeutic possibilities for both Gram-positive and Gram-negative, with the possibility for Gram-positive also of long-acting drugs. At the same time, several and new therapeutic options are available to address MDR Gram-negative microorganisms. These antibiotic therapies allow us to address the issues concerning antimicrobial resistance. Although we have different and new therapeutic options to deal with MDR germs, it is nevertheless necessary to underline the importance of the prevention of and the control of infections in this setting. In fact, prevention and infection control are the main strategies to be able to counter this problem. Therefore, if on the one hand we have various therapeutic possibilities available to manage the infection, at the same time, the implementation of preventive measures and of infection control represent the strategies for the management of surgical site infections.
